# Fibroepithelial Polyp of the Vagina With Torsion: A Difficult Diagnosis Based on Clinical and Morphological Findings of the Vaginal Lesion

**DOI:** 10.7759/cureus.55157

**Published:** 2024-02-28

**Authors:** Efthymia Thanasa, Anna Thanasa, Gerasimos Kontogeorgis, Ektoras-Evangelos Gerokostas, Ioannis-Rafail Antoniou, Athanasios Chasiotis, Emmanouil M Xydias, Apostolos C Ziogas, Evangelos Kamaretsos, Ioannis Thanasas

**Affiliations:** 1 Department of Histology, Faculty of Health Sciences, Aristotle University of Thessaloniki, Thessaloniki, GRC; 2 Department of Anatomy, Faculty of Health Sciences, Aristotle University of Thessaloniki, Thessaloniki, GRC; 3 Department of Obstetrics and Gynecology, General Hospital of Trikala, Trikala, GRC; 4 Department of Obstetrics and Gynecology, Limassol General Hospital, Limassol, CYP; 5 Department of Obstetrics and Gynecology, EmbryoClinic In Vitro Fertilization (IVF) Unit, Thessaloniki, GRC; 6 Department of Obstetrics and Gynecology, University of Thessaly, Larissa, GRC

**Keywords:** vaginal fibroepithelial polyp, torsion, clinical findings, histological diagnosis, surgical treatment, case report

## Abstract

Vaginal fibroepithelial polyps are rare benign tumors of the mucosa of the anterior vaginal wall. In extremely rare cases, they may originate from the posterior vaginal wall or be complicated by torsion. Our case concerns a 63-year-old patient who presented to the gynecology outpatient clinic of the General Hospital of Trikala with minor vaginal bleeding. On vaginal examination, a large pedunculated painless hemorrhagic polypoid mass was noticed, originating from the posterior vaginal wall. A torsion of the pedunculated vaginal tumor was suspected, leading to its surgical excision with clear resection margins. Due to extensive tissue necrosis, accurate histological identification of the vaginal neoplasm was not possible. Histological examination excluded vaginal malignancy. Based predominantly on the clinical and morphological features of the vaginal lesion, a diagnosis of vaginal fibroepithelial polyp with torsion was made, acknowledging its limitations. The patient was discharged from the clinic the same afternoon following the surgery. Three months later, no recurrence of the lesion in the vaginal wall was noted. Following the case presentation, this paper provides a brief literature review of this rare entity, focusing on the diagnostic and therapeutic approaches.

## Introduction

Vaginal neoplasms (benign or malignant) are rare [1]. Primary vaginal cancer represents a rare malignancy of the female reproductive tract [2]. It is estimated to comprise 10% of all malignant neoplasms affecting the vagina [3] and 1-2% of all gynecological cancers [4]. Although primary vaginal cancer typically occurs in postmenopausal women, there has been an increase in its incidence among young women in recent years, particularly those with persistent high-risk human papillomavirus infection [5]. Also, papillomas, hemangiomas, fibroepithelial polyps, and leiomyomas are uncommon benign tumors of the vagina, typically growing on the anterior vaginal wall. Vaginal tumors originating from the lateral or posterior wall are even rarer [6].

Vaginal fibroepithelial polyps are benign polypoid mucosal lesions with a core of connective tissue covered by squamous epithelium [7]. The pathogenetic mechanism has not been fully elucidated to date. Hormonal stimulation appears to be an important predisposing factor. Vaginal fibroepithelial polyps typically occur during pregnancy and may resolve after delivery. They can also be associated with hormonal replacement therapy or tamoxifen therapy [8]. Fibroepithelial polyps are usually located on the anterior vaginal wall, near the vaginal opening. In rare cases, a significant increase in size may result in prolapse of the polyp outside the vagina [9]. In our case, the fibroepithelial polyp originated from the posterior vaginal wall, at the junction between the posterior vaginal vault and the lateral left vaginal wall, and was associated with torsion of the vascular pedicle, which appears to be a unique clinical report in the English literature.

This paper highlights the rarity of vaginal fibroepithelial polyps, especially when originating from the posterior vaginal wall and complicated by torsion, which may present differential diagnostic challenges with malignant lesions of the vaginal wall. Concurrently, it emphasizes the necessity of surgically excising the lesion with clear resection margins, conducting histological examinations, and following up with these patients for early detection of disease recurrence.

## Case presentation

A 63-year-old patient presented at the gynecology outpatient clinic of the General Hospital of Trikala complaining of mild vaginal bleeding persisting for approximately five days. The vaginal bleeding was not accompanied by abdominal pain or pain localized in the vagina or external genitalia. The patient had been menopausal for about 15 years, and according to her, there had been no incident of vaginal bleeding during that time. The patient's gynecological screenings had not been regular. The last Pap smear test, which had normal findings, was conducted five years ago. The patient had two vaginal deliveries in her obstetric history. She did not report any hematological disorders in her personal history. She did report hypothyroidism and arterial hypertension, both of which were well-regulated with appropriate medication. The patient did not receive hormone replacement therapy.

**Figure 1:**
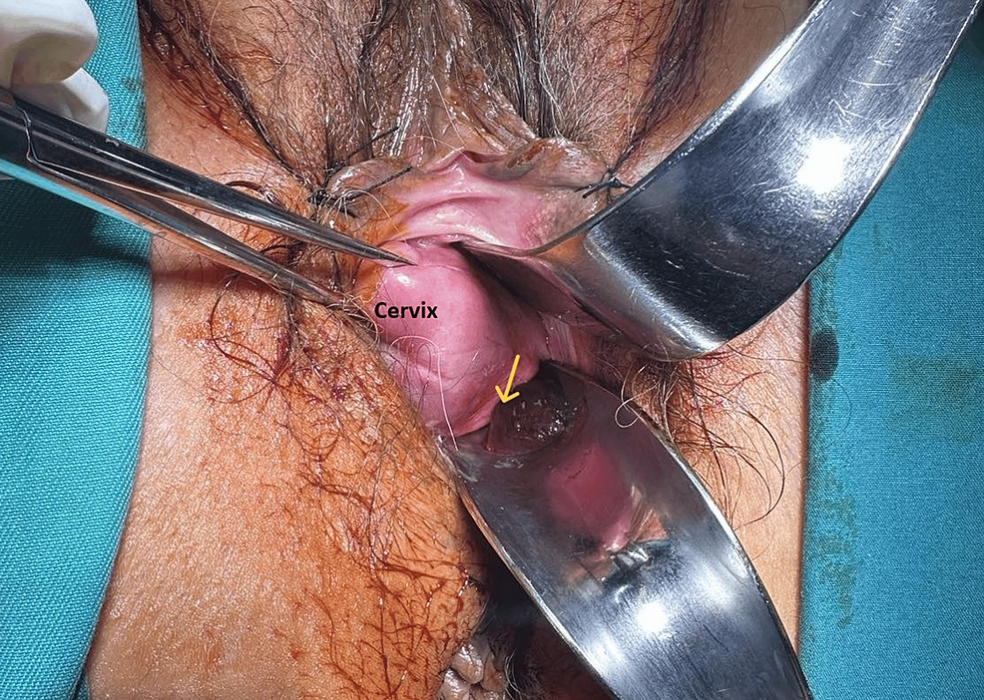
Vaginal examination of a pedunculated fibroepithelial polyp The origin of the neoplasm from the posterior vaginal wall (yellow arrow) with torsion of the pedicle is not a common presentation.

On vaginal examination using a speculum, mild vaginal bleeding was found to be caused by a large pedunculated polypoid mass, dark in color, originating from the posterior vaginal wall at the junction between the posterior vaginal vault and the left lateral vaginal wall (Figure 1).

**Figure 2:**
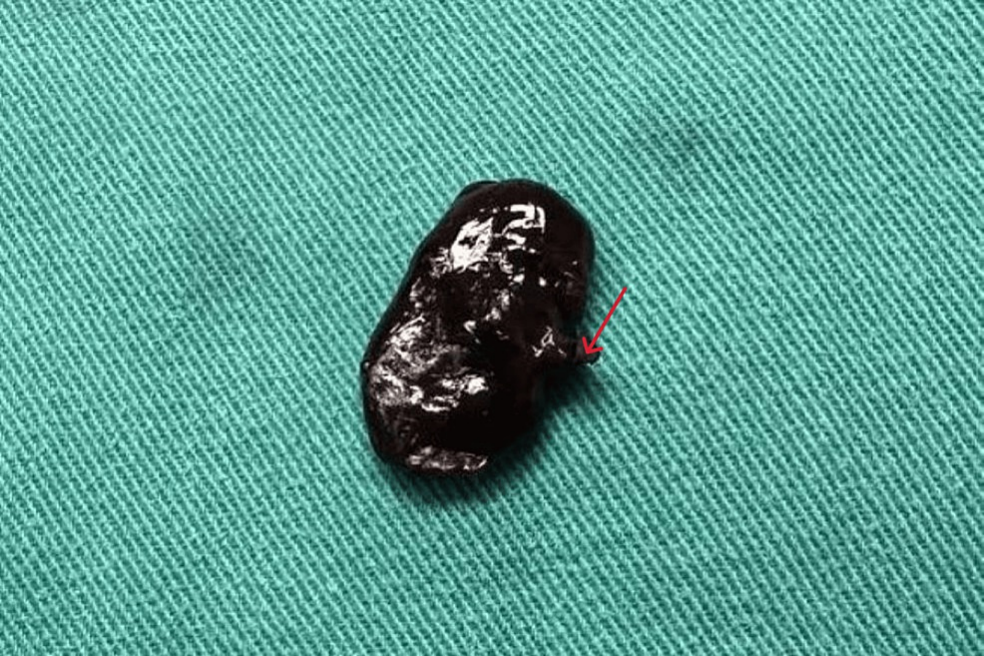
Surgical specimen of the vaginal fibroepithelial polyp Complete necrosis of the neoplasm and the presence of a vascular pedicle (red arrow) demonstrate torsion of the vaginal tumor.

Transvaginal ultrasound revealed no abnormal findings from the uterine corpus, endometrium, or adnexa. A Pap smear test was performed, and the cytological findings were normal. The blood tests revealed: hematocrit (Ht) 38.6%, hemoglobin (Hb) 13.4 g/dL, platelets (PLT) 232 x 103/mL, white blood cells (WBC) 6.41 x 103/mL, and neutrophils (NEUT) 62.6%. Coagulation and biochemical tests showed no abnormal findings. Based on the clinical findings, a polypoid lesion of the vagina with torsion was suspected, which was surgically excised with clear resection margins (Figure 2).

**Figure 3:**
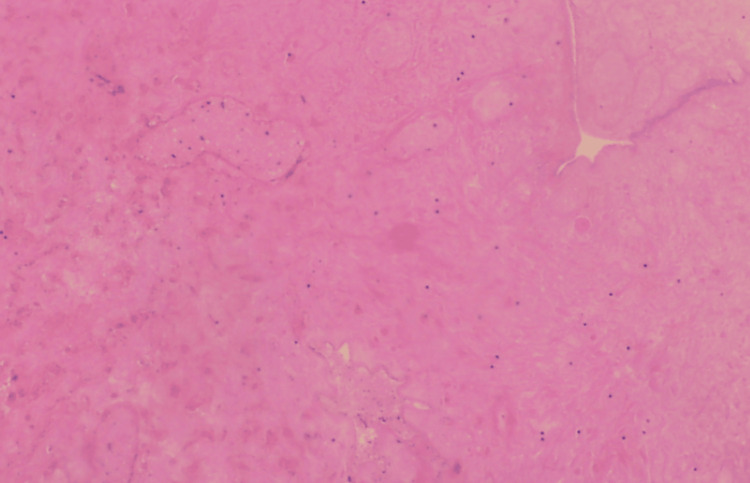
Histological image of the pedunculated vaginal fibroepithelial polyp A hemorrhagic necrotic subcellular lesion is depicted, with few areas of spindle-shaped cells without atypia and with dilated congested blood vessels, indicative of torsion.

Upon histological examination, macroscopic findings revealed a polypoid lesion with a dark color and a maximum diameter of 3 cm. Microscopically, a hemorrhagic necrotic sub-cellular neoplasm was observed, in a few locations of which spindle-shaped cells with small nuclei without atypia were seen. No evidence indicative of suspected malignancy was observed (Figure 3).

Due to the extensive necrosis, precise identification of the neoplasm was not feasible. The patient was discharged from the obstetrics and gynecology clinic of our hospital on the same afternoon following the surgery. Three months later, no signs of recurrence of the lesion in the vaginal wall were observed. The patient was recommended to undergo annual follow-up at the gynecology outpatient clinic of the General Hospital of Trikala.

## Discussion

Vaginal fibroepithelial polyps pose significant diagnostic challenges due to their rarity, atypical clinical manifestations, and diverse histological characteristics. Hypercellularity and cytologic atypia may contribute to misdiagnosing them as malignant connective tissue lesions [10]. Vaginal fibroepithelial polyps are uncommon clinical entities that can present either as small, asymptomatic tumors or as large, symptomatic ones, depending on their size. Clinical findings associated with vaginal fibroepithelial polyps may include vaginal bleeding or bloody vaginal discharge, dyspareunia, obstruction of the genitourinary tract, a sensation of pressure and heaviness in the vagina, and the presence of tissue protruding from the vagina [7,11]. In our patient, the presence of a pedicle combined with the increased size of the polypoid lesion resulted in the torsion of the tumor and the occurrence of painless vaginal bleeding. Paradoxically, our patient did not complain of pelvic pain. This is most likely attributed to the thinness and reduced vascularity of the vascular pedicle of the fibroepithelial polyp, which prevented ischemia and necrosis of the vaginal neoplasm from eliciting pain. In any case, vaginal fibroepithelial polyps should be differentiated from malignant neoplasms of the vagina, such as sarcoma botryoides, rhabdomyosarcoma, and mixed mesodermal tumors [12]. Furthermore, vaginal leiomyomas, although extremely rare, should be included in the differential diagnosis of vaginal fibroepithelial polyps [13].

The role of imaging modalities in the preoperative diagnosis of fibroepithelial polyps of the vagina has not been extensively evaluated. Although magnetic resonance imaging findings have been documented in several cases of fibroepithelial polyps originating from various organs, including the vulva, magnetic resonance imaging findings during the preoperative assessment of fibroepithelial polyps of the vagina have been reported in only one study in the English literature. More specifically, in 2022, Ogura et al. presented the findings of magnetic resonance imaging of a vaginal fibroepithelial stromal polyp in a postmenopausal woman and concluded that magnetic resonance imaging may be a useful non-invasive method for the preoperative diagnosis of vaginal fibroepithelial stromal polyps and their differentiation from malignant lesions of the vagina [14].

However, despite the suspicion that may be raised by the pelvic examination and the MRI findings, the diagnosis of vaginal fibroepithelial polyps is confirmed by histological examination. Microscopically, the fibroepithelial stromal polyp consists of the central fibrovascular core, stroma, and the overlying benign squamous epithelium [15]. The typical feature of these lesions is the presence of abundant edematous or fibrous stroma-containing stromal cells. These stromal cells, which exhibit mild nuclear features, range from spindle-shaped to stellate cells, with some multinucleated forms. The squamous epithelium ranges from normal to hyperplastic. Additionally, the stromal cells of a fibroepithelial polyp consistently seem to react to desmin, estrogen receptors, progesterone receptors, and sometimes to smooth muscle actin [16]. In our patient, the presence of a vascular pedicle in combination with the increased dimensions of the polypoid lesion led to the torsion of the vaginal tumor and the manifestation of vaginal bleeding. The extensive tissue necrosis observed within the neoplasm posed significant challenges in the differential diagnosis, including distinguishing it from vaginal malignancy or vaginal leiomyoma. The existence of malignancy was excluded histologically. Immunohistochemistry was not feasible due to necrosis of the vaginal neoplasm. Based on the macroscopic features of the tumor, the diagnosis of a fibroepithelial polyp of the vagina was established rather than vaginal leiomyoma.

The treatment of vaginal fibroepithelial polyps is surgery. Immediate planned surgery is the only treatment option, especially in those cases where the vaginal lesion is associated with ischemia, necrosis, and bleeding, as in our patient. Surgical excision of the polyp with clear resection margins is considered curative. If the resection of the lesion is incomplete, a recurrence of the disease is likely [17]. In cases where magnetic resonance imaging confirms the benign nature of the fibroepithelial polyp, resection of the lesion from the vaginal wall could be accomplished with an electric scalpel at the root of the stalk without necessitating a radical surgical margin [14]. Due to the risk of recurrence, continuous follow-up with regular gynecological examinations is necessary [9].

Limitations

We report a case of a vaginal polypoid lesion with torsion that presented with postmenopausal metrorrhagia. Complete necrosis of the vaginal neoplasm, complicated by torsion, did not enable a reliable histological diagnosis. Histologically, a hemorrhagic necrotic subcellular neoplasm was observed, with few spindle-shaped cells with small nuclei without atypia. Due to the extensive necrosis of the tumor, precise identification of the fibroepithelial vaginal polyp and its differentiation from the vaginal leiomyoma was not feasible. The localization of a fibroepithelial polyp on the posterior vaginal wall is extremely rare. Vaginal leiomyomas are also exceedingly rare, with approximately 300 cases reported in the literature to date [18]. Immunohistochemistry was not possible due to the complete necrosis and damage observed in the examined vaginal neoplasm. The diagnosis of vaginal fibroepithelial polyp with torsion in our patient was primarily based on the clinical and morphological features of the vaginal lesion (shape, size, color, presence of pedicle), the exclusion of malignancy of the vagina as revealed by histological examination, and the rarity of vaginal leiomyomas, particularly those originating from the posterior vaginal wall.

## Conclusions

Vaginal fibroepithelial polyps are rare benign tumors primarily found on the anterior vaginal wall. Localization on the posterior or lateral vaginal wall is even rarer. The torsion of a pedunculated vaginal fibroepithelial polyp diagnosed in our patient represents a unique case reported in the English literature. In such cases, extensive necrosis and damage to the surgical specimen make histological diagnosis difficult. Diagnosis can rely on the clinical and morphological features of the vaginal lesion. Follow-up with these patients is considered essential for the early detection of disease recurrence.
